# KiMoSys 2.0: an upgraded database for submitting, storing and accessing experimental data for kinetic modeling

**DOI:** 10.1093/database/baaa093

**Published:** 2020-11-28

**Authors:** Hugo Mochão, Pedro Barahona, Rafael S Costa

**Affiliations:** Departamento de Informática Faculdade de Ciências e Tecnologia, Universidade NOVA de Lisboa Campus de Caparica, 2829-516, Caparica, Portugal; NOVA LINCS, Dept. Informática Faculdade de Ciências e Tecnologia, Universidade NOVA de Lisboa Campus de Caparica, 2829-516, Caparica, Portugal; LAQV-REQUIMTE, Departamento de Química, Faculdade de Ciências e Tecnologia, Universidade NOVA de Lisboa Campus de Caparica, 2829-516, Caparica, Portugal and; IDMEC, Instituto Superior Técnico, Universidade de Lisboa, Av. Rovisco Pais 1, 1049-001, Lisboa, Portugal

## Abstract

The KiMoSys (https://kimosys.org), launched in 2014, is a public repository of published experimental data, which contains concentration data of metabolites, protein abundances and flux data. It offers a web-based interface and upload facility to share data, making it accessible in structured formats, while also integrating associated kinetic models related to the data. In addition, it also supplies tools to simplify the construction process of ODE (Ordinary Differential Equations)-based models of metabolic networks. In this release, we present an update of KiMoSys with new data and several new features, including (i) an improved web interface, (ii) a new multi-filter mechanism, (iii) introduction of data visualization tools, (iv) the addition of downloadable data in machine-readable formats, (v) an improved data submission tool, (vi) the integration of a kinetic model simulation environment and (vii) the introduction of a unique persistent identifier system. We believe that this new version will improve its role as a valuable resource for the systems biology community.

**Database URL:**  www.kimosys.org

## Introduction

Sharing experimental data has major advantages for the scientific community. With the amount of biological data produced increasing each year, structured databases are a crucial tool to store, share and maintain data, improving quality and reproducibility (1). Being able to have this information aggregated in a single discipline-specific repository could spare a lot of time.

Experimental data take a big part in constructing kinetic models of biological systems. A kinetic model construction is mainly composed of four steps that can proceed iteratively: (i) model calibration/building (the rate reactions are usually represented mathematically by ordinary differential equations), (ii) simulation, (iii) model validation and analysis and finally (iv) model applications (2). The estimation/identification of unknown parameters is a critical task of model building, and when fitting a kinetic model from data or evaluating model predictions (e.g. in Chassagnole *et al.* (3), Khodayari *et al.* (4) and Mannan *et al.* (5)), a curated repository with metabolomics, fluxomics and proteomics data will be essential (6). Alternatively, curated databases such as BRENDA (7) and SABIO-RK (8) provide relevant kinetic information (rate equations and experimentally derived kinetic parameters) available in the literature, which can support the model construction or extension, while repositories like BioModels (9) and JWS Online (10) stored Systems Biology Markup Language (SBML) (11) models and their properties. To complement data/model annotations and information, specific database identifiers (e.g. KEGG, ChEBI, UniProt and NCBI) associated with each biochemical entity are also important.

KiMoSys database (12) aims to aggregate and provide published experimental data (steady state and/or dynamics of metabolite concentrations, reaction fluxes and enzyme measurements) into a single location, which can be connected to the corresponding associated kinetic model(s) (i.e. the data set that is used to build and/or evaluate the model). The public repository is focused on data dissemination and collaboration procedures to facilitate data reuse by the systems biology modelers and experimentalists. Currently, KiMoSys is a recommended data repository by a number of leading scientific journals including PLOS (https://plos.org), Scientific Data (https://www.nature.com/sdata/), GigaScience (https://academic.oup.com/gigascience) and BioMed Central (https://www.biomedcentral.com). Similarly, it is also registered by other organizations, such as the Data Citation Index on the Web of Science (13), FAIRsharing (14) and re3data (15).

One major problem regarding the aggregation of data from multiple laboratories/sources is the different nomenclature, conditions and protocols adopted by each research group. Moreover, quantitative data are usually difficult for a direct comparison between the simulated and measured data because of the formats and units used. Thus, it is essential that the experimental data and models are stored in a structured format and according to standards well known by the community. For experimental data, the Minimum Information standards, created in 2008, is used and called the Minimum Information for Biological and Biomedical Investigations (MIBBI) project (16). For instance, the Minimum Information About a Microarray Experiment (MIAME), part of MIBBI, was created to describe microarrays with the minimum information possible (17). Metadata is also a very important aspect to take into account when storing experimental data. Experience details, description and other important information can help to better understand the experimental set-up and procedure. The ISA (Investigation–Study–Assay) format is an open-source tool that allows producing detailed metadata information, enhancing the reproducibility and reusability of the data (18). For storing mathematical models, SBML is the most common standard, and it is based on XML. CellML (19), also based on XML, Biological Pathway Exchange (20) and MATLAB are other popular languages to store and deal with computational models in systems biology. To respond to the challenge of data discovery, the DataCite consortium (21) provides a service to assign digital object identifiers (DOIs) as a standard persistent identifier for data citation.

Scientific journals have been making an effort to encourage researchers to make data shareable and public, endorsing public data repositories (22). They advise the repositories to follow certain specifications of how these should work, the FAIR (Findable, Accessible, Interoperable and Reusable) Principles (23) being the main reference. McQuilton *et al.* propose criteria that strengthen data FAIRness (24). By agreeing to these specifications, the likelihood of repositories being recommended or certified by journals and publishers increases. However, as shown by Husen *et al.* (22) in 2017, the process of certification is still in maturation, as less than 6% of the recommended repositories (evaluated in that study) had a certification.

In this new release, KiMoSys upgraded its capabilities in this direction and was empowered with several new features, especially focused on data visualization, intuitive and user-friendly web interfaces, more options to manage and download data formats, a new simulation environment for the models and the introduction of a DOI system. Furthermore, new publicly available data sets and associated kinetic models were integrated into the database.

The paper is structured as follows: the section “Methods and Implementations” provides information about the methods and technologies used; the section “Results” describes the new features and the advantages of having them; the final remarks are present in the section “Concluding Remarks.”

## Methods and Implementations

The initial version of KiMoSys was built using the Ruby on Rails framework (https://rubyonrails.org), which uses the MVC (Model–View–Container) pattern, and is a powerful tool to build websites in a short amount of time (25). It was necessary to make an extensive update of the code since this software has received several major changes and upgrades since KiMoSys was first deployed. Ruby uses ‘gems’, which are external libraries focused on specific functionalities. While some of these gems are maintained officially by Ruby on Rails, many others are developed by different companies or by the Rails community.

To improve the user interface, by making it more accessible and user-friendly, Bootstrap framework (https://getbootstrap.com) was introduced. Being the most popular tool in the world concerning HTML, CSS and Javascript (26), it provides clean and appealing interfaces.

The repository table in which all data sets can be searched and filtered in multiple ways is handled by DataTables (https://datatables.net). This software has a large number of extensions that can be used, one being SearchPanes (https://datatables.net/extensions/searchpanes/) that has the ability to create a filter panel. This panel is intuitive and easy to use, making it faster to search for the wanted data.

In order to preview, modify or create plots from XML, CSV, SBML and XLSX files, it is necessary first to parse them. To achieve this, many gems are used. To preview XML, the file is first parsed using REXML (https://github.com/ruby/rexml), and then Google’s prettify (https://github.com/googlearchive/code-prettify) makes an understandable display of it, with line numbers and different colors to highlight the code. Nokogiri (https://nokogiri.org) is used to parse SBML files, while roo (https://github.com/roo-rb/roo) parses CSV and XLSX. Finally, rubyXL is involved in the process of creating new XLSX files.

Regarding data visualization, chartkick (https://chartkick. com) was the chosen library. Having the impressive ability to produce plots with just a line of Ruby code, this gem is also able to use three different chart libraries: Chart.js (https://www.chartjs.org), Highcharts (https://www.highcharts.com) and Google Charts (https://developers.google.com/chart).

The simulation of kinetic models is processed by COPASI software (27), using its Python language bindings. These provide an extensive set of functionalities regarding the simulation of computational models. KiMoSys 2.0 uses the time-course and steady-state simulation features/tasks. Thus, each simulation is achieved by running a Python script, which generates an output that is used to build the plots and tables.

With the objective to include Javascript libraries, Webpacker (https://github.com/rails/webpacker) was introduced. Using Webpacker, it is possible to add these Javascript libraries via Yarn (https://yarnpkg.com). Bootstrap and DataTables were imported to the project using this tool.

KiMoSys uses an SQLite3 database (https://www.sqlite.org/index.html), and Active Storage (https://github.com/rails/rails/tree/master/activestorage) is responsible to manage the storing of files.

Data and models are published as citable DOIs, registered at DataCite through FCT-NOVA University Lisbon (https://www.fct.unl.pt) services.

Other important libraries used are as follows:

- activeadmin (https://activeadmin.info), which provides an admin page where data can be easily managed.
- devise (https://github.com/heartcombo/devise), that is responsible for user registrations, logins and sessions.
- Google Recaptcha (https://www.google.com/recaptcha/intro/v3.html), to prevent bots from creating accounts.
- cancancan (https://github.com/CanCanCommunity/cancancan), which deals with user permissions.
- impressionist (https://github.com/charlotte-ruby/impressionist), which handles the counting of pages views and downloads.
- SimpleForm (https://github.com/heartcombo/simple_form), a tool that is able to generate forms with Bootstrap.
- MathJax (https://www.mathjax.org), which helps transforming raw mathematics equations into clear and readable images.

## Results

The main architecture of KiMoSys has been retained, as provided in the original publication. However, the platform has undergone significant changes to implement the new functionalities and features, described next.

### New queries and improved web interface

**Figure 1.**
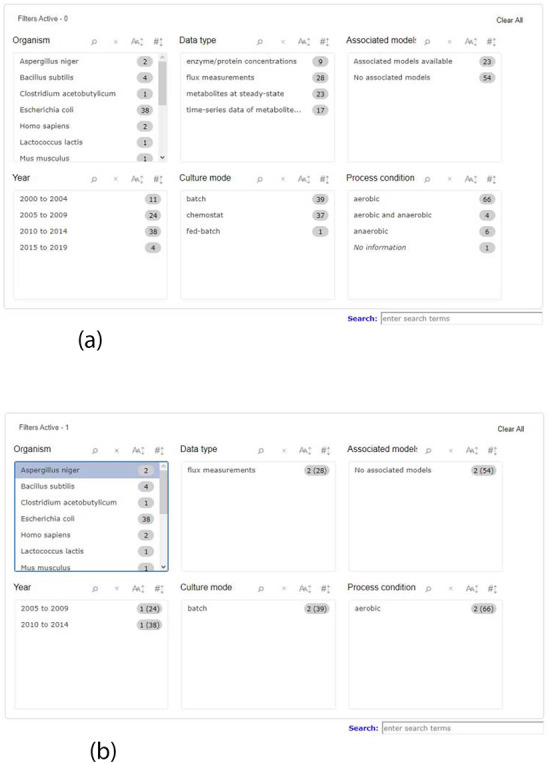
Screenshot of the new filter panel and its interactions. Ahead of each item, there is a number that represents its amount present in the database. (a) A filter panel for the repository table. (b) An example of the new filter panel after the selection of an item; the panel refreshes according to the selected filter and the remaining available options. The numbers are updated: the value outside the parentheses is the remaining amount of data entries that exist according to the filters selected, while the value inside the parentheses is the total number present in the database.

When searching for data, it is necessary to have a wide range of queries, covering the most relevant data attributes, therefore facilitating the process to the user. To meet this demand, KiMoSys 2.0 introduced a completely new filter query panel, offering different pre-structured queries/filters, allowing, for example, the selection of an organism name, data type or metadata information, such as year of publication where the data are described or the culture mode. There are six main filter blocks that the user can select, as shown in Figure 1a. The user can filter a query to focus only on a specific characteristic, refreshing the panel according to the options that are still left, relative to the one that was clicked, providing a better user experience as depicted in Figure 1b. Users can choose multiple filters in the same box, as well as in different ones. The items displayed in each box can be ordered alphabetically or numerically. Additionally, it is also possible to combine the free text search box available in the previous version, with the new filters. The search box allows users to search each metadata field available. The option to search the data and model elements (metabolite, reaction, protein) by standard cross-references ChEBI, KEGG or UniProt IDs is also available in KiMoSys 2.0.

**Figure 2.**
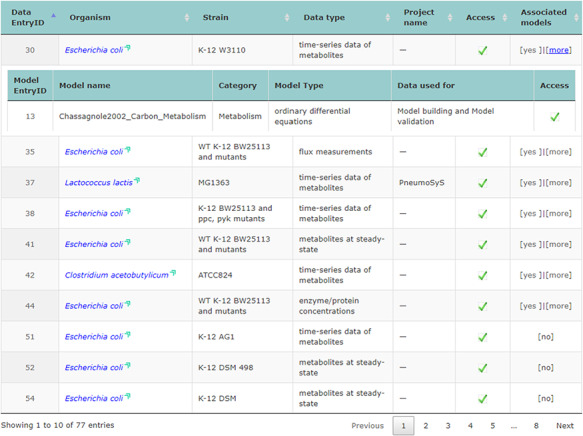
Screenshot of the main repository table.

The main repository table was refined for a more dynamic appearance. For user convenience, table entries with data that have connected associated models can be expanded and collapsed to display information related to them (displayed in Figure 2).

**Figure 3.**
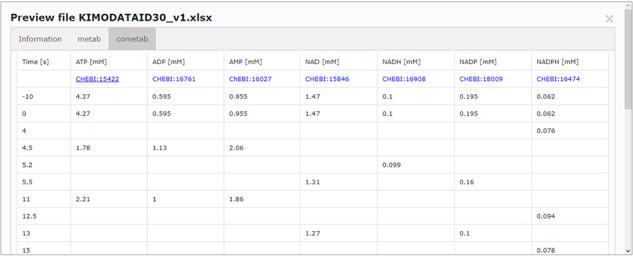
Screenchot depicting a data file preview (example for DataEntry ID 30). Here, the user can navigate through each tab.

**Figure 4.**
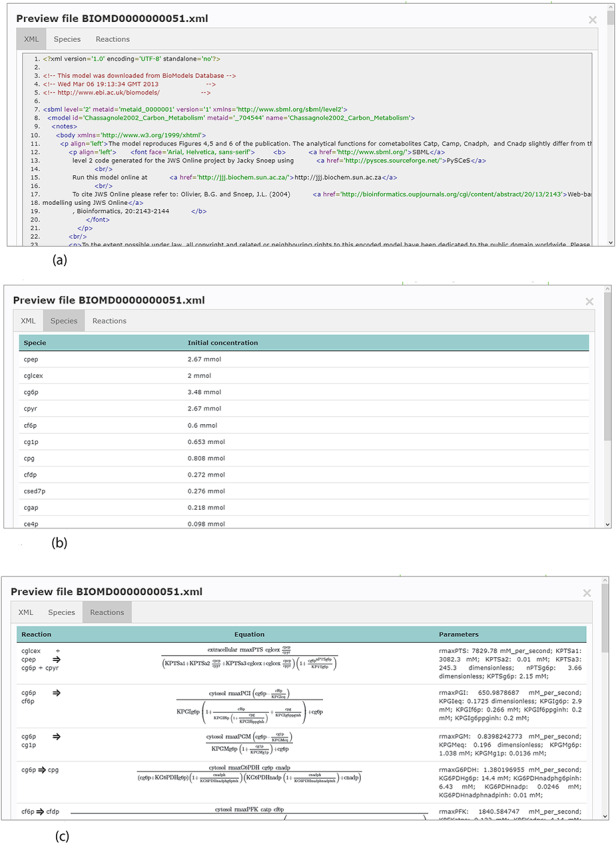
Example of an SBML model file preview (Model EntryID 13). (a) Snapshot of the XML file preview. (b) Screenshot for the model species and initial concentrations. (c) Screenshot of the model reactions, rate equations and kinetic parameters.

Accessing data displayed in a clear and understandable way, without having to download it, provides a more friendly and engaging environment to the user. KiMoSys new version allows the preview of the CSV, XLSX files (Figure 3) and SBML, XML files (Figure 4) for data sets and models, respectively. Users can navigate through the XLSX tabs, just like in an Excel file, and below for each table, a plot of the data is displayed. The metabolite, flux and protein entities are accompanied by cross-links to external resources (ChEBI, KEGG and UniProt). The SBML file has color highlighting on the code to help the user have a better understanding of it (Figure 4a). Additionally, the species (Figure 4b), kinetic rate equations and parameters (Figure 4c) of a kinetic model are also provided to preview. Unfortunately, for the models stored in KiMoSys as executable code (e.g. MATLAB), the preview option is unavailable.

As data and metadata citation is important to make it more Accessible and Reusable, an easier way was implemented to cite the data and models, with some generic citation formats, such as APA, MLA, ISO-690 and BibTex, and a copy to the clipboard.

Clear and intuitive interfaces are essential to improve the user experience. To achieve this, Bootstrap (https://getbootstrap.com) was introduced. Several minor interface changes were done, such as font size or boxes layout. The most significant update was made in forms that now use the Simple Form ‘gem’ (https://github.com/heartcombo/simpleform), which provides an easy tool to create Bootstrap forms Submitting data, registering a new account, or navigate through the website can now be easier to the users.

**Figure 5.**
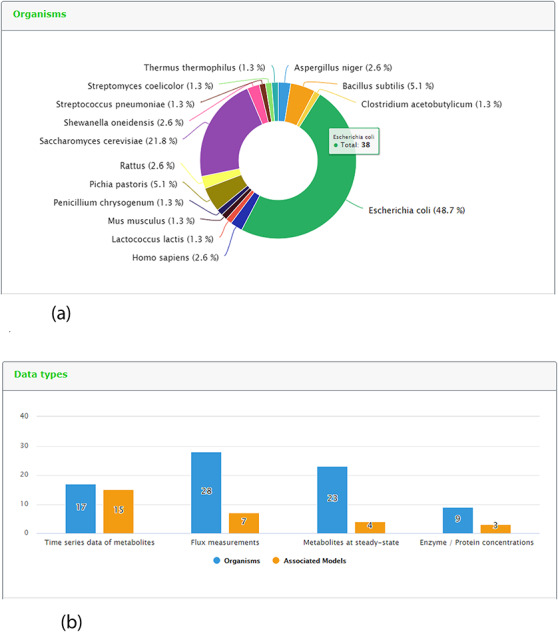
Representative screenshots from the new content statistics page tab. (a) Overview of organisms statistics. (b) Overview of data type and associated model statistics.

One new page tab has been created for the present KiMoSys release. The content statistics tab, evidence by organisms and data type (and its associated models) are shown in (Figure 5a) and (Figure 5b), respectively. In this update, the data deposited in KiMoSys were expanded with significantly new public experimental data sets and associated kinetic models. The repository was upgraded and manually curated from over 50 bibliographic references. From these, one submission has been accompanied by corresponding journal submission (28). This example shows a system biology work (with experimental data sets) where the data have been submitted to the KiMoSys repository (with the data set identifier Data EntryID 91) and cited in the original paper (28). Currently, the complete set of experimental data and associated kinetic models in KiMoSys contains 77 public data sets and 23 associated kinetic models entries (compared with 36 Data EntryIDs and 12 associated models, in April 2014). This information was manually extracted and collected from published scientific articles and added to the KiMoSys database. We hope that in the future other research groups (modelers but also experimentalists) can use this information for additional studies. It has 28 ‘metabolic fluxes’ records (36%) that are linked to 7 associated kinetic models; 17 ‘time-series metabolites’ records (22%) linked to 15 associated kinetic models; and 23 ‘steady-state metabolites’ records (30%) linked to 4 models. A total of 9 ‘protein levels’ (12%) data sets that are linked to 3 kinetic models are also contained in the repository. *Escherichia coli* (49%) is by far the organism with the highest number of identifiers (38 Data EntryIDs and 11 Model EntryIDs). Apart from *E. coli*, the second most represented type of organisms in KiMoSys is *Saccharomyces cerevisae* (22%). This should come as no surprise since these are two well-studied organisms described in the literature. Other organisms (more than 10) including *Pishia pastoris, Lactococcus lactis* and *Aspergillus niger* are also presented.

### Implementation of data visualization

**Figure 6.**
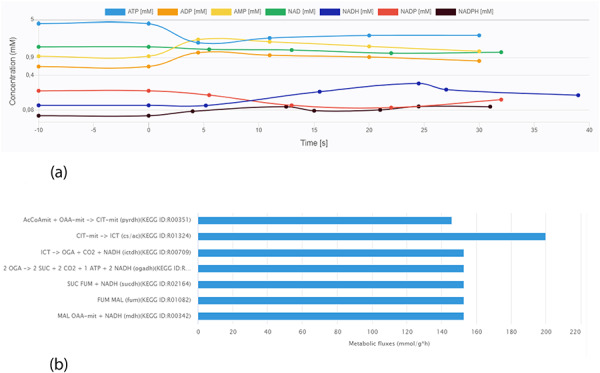
Screen images from different data types that the user can preview from the data sets. (a) Example of time-course data for the Data EntryID 30. (b) Example of flux distribution for the Data EntryID 73.

Data visualization is crucial for a more intuitive interpretation of experimental results. A key feature of the KiMoSys database is the introduction of a visualization service for the data sets stored. The different viewing options (for metabolites’ concentration over time or in steady-state and flux distributions) allow users for a better perception of the data. As an example, Figure 6 displays two plots from different data files.

The ability to preview data files, without having to download them and use other tools to create a plot can be very helpful, sparing time to the user and facilitating the data set interpretation. Moreover, providing the user with an overview of the associated models (specie concentrations, kinetic rate equations and parameters) in a human-readable way is also important for a better perception of the models’ characteristics.

### More downloadable data formats

As metadata is considered fundamental to describe data, it is very important to have it available to download in diverse formats. In addition to the search results view and an improved web interface, users can now export the data and/or model metadata. It can be downloaded in three formats: RDF (Resource Description Format), XML and plain text. This feature achieves the Interoperable FAIR Principle, which is focused on combining data by humans and machines in standardized formats, while also enforcing data Reusability since it can be processed using computational methods (such as document creation from XML or RDF files). Furthermore, statistics about the total number of downloads and accesses are provided for each data set and associated model record.

### Improved data submission tool

In KiMoSys, there are two main types of data submission: a quick submit that does not require user registration, and a more elaborate one, requesting detailed information. In this release, an improved electronic data-submission tool is offered. In the original version, it was necessary to fill an Excel file with the data and metadata, and then fill an online form with the same metadata, which was not user-friendly. Now it is not necessary to fill the Excel spreadsheet with the metadata. When the user submits the online form, the metadata is automatically aggregated to the file with the data set. The data version present in the file is also automatically updated when data are modified. This new tool is expected to increase the rate at which data are deposited to the KiMoSys database by the community and the quality of the submitted information.

### New simulation environment

**Figure 7.**
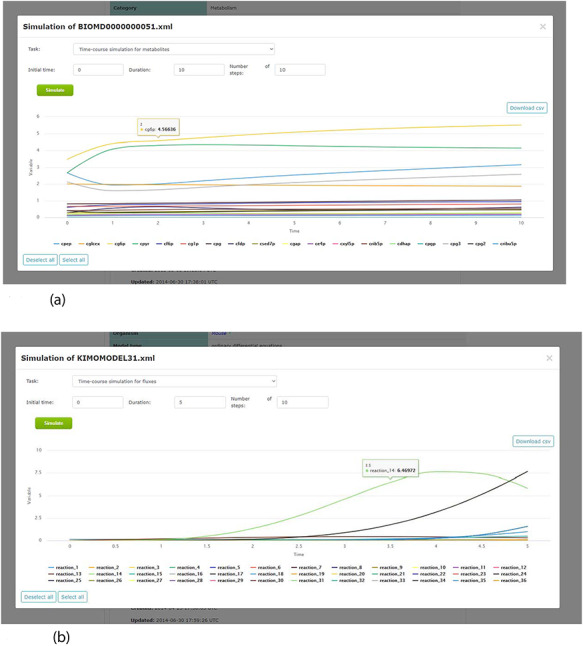
Screenshots of the simulation web interface for two different associated models stored in KiMoSys. The CSV file is available to download the simulated data. (a) Example of metabolites time-course simulation with the Model EntryID 13. (b) Time profiles for the reaction fluxes simulation obtained with the Model EntryID 31.

By including a simulation environment for kinetic models, using COPASI software, KiMoSys 2.0 introduced new options to facilitate the use of the associated kinetic models. The researchers can use the web-based interface to explore metadata, and perform simulations (time-course and/or steady-state) for the stored kinetic models (see examples in Figure 7), by clicking the ‘Simulate’ button. This can be done only for the models in SBML, the universal standard format in systems biology. Users can also export the simulation data in CSV format.

### New unique persistent identifier system

In order to be considered a FAIR compliant resource, KiMoSys 2.0 introduced a DOI for each Data/Model EntryID, which is in line with the FAIR Data Principle requests (23). Data and model records available in the database are now published as DOIs and registered at DataCite. For new data/model entities submitted to the KiMoSys database and approved by the KiMoSys team, a citable DOI is assigned as a permanent reference. Thus, in this updated version, the DOI system is currently available to provide persistent, unique and citable data, conceding KiMoSys the Findable FAIR Principle.

## Concluding remarks

KiMoSys 2.0 is a significant update to the original version. It provides new tools for data visualization, models simulation, (meta)data search, machine-readable download formats and data submission process, while also introducing a new web interface that can improve the user experience. These new features are in accordance with the FAIR standards, and along with the introduction of a unique persistent identifier system make KiMoSys a Findable repository. In addition, the number of records and source publications consulted has increased. We believe that KiMoSys 2.0 will be very helpful to the systems biology modeling community. In the future, KiMoSys will be routinely maintained as the collection/curation of data (and associated kinetic models) is ongoing.
